# The Mediterranean Benthic Herbivores Show Diverse Responses to Extreme Storm Disturbances

**DOI:** 10.1371/journal.pone.0062719

**Published:** 2013-05-07

**Authors:** Jordi F. Pagès, Alessandro Gera, Javier Romero, Simone Farina, Antoni Garcia-Rubies, Bernat Hereu, Teresa Alcoverro

**Affiliations:** 1 Centre d’Estudis Avançats de Blanes (CEAB-CSIC), Blanes, Spain; 2 Departament d’Ecologia, Facultat de Biologia, Universitat de Barcelona, Barcelona, Spain; 3 Nature Conservation Foundation, Mysore, India; The Australian National University, Australia

## Abstract

Catastrophic storms have been observed to be one of the major elements in shaping the standing structure of marine benthic ecosystems. Yet, little is known about the effect of catastrophic storms on ecosystem processes. Specifically, herbivory is the main control mechanism of macrophyte communities in the Mediterranean, with two main key herbivores: the sea urchin *Paracentrotus lividus* and the fish *Sarpa salpa*. Consequently, the effects of extreme storm events on these two herbivores (at the population level and on their behaviour) may be critical for the functioning of the ecosystem. With the aim of filling this gap, we took advantage of two parallel studies that were conducted before, during and after an unexpected catastrophic storm event. Specifically, fish and sea urchin abundance were assessed before and after the storm in monitored fixed areas (one site for sea urchin assessment and 3 sites for fish visual transects). Additionally, we investigated the behavioural response to the disturbance of *S. salpa* fishes that had been tagged with acoustic transmitters. Given their low mobility, sea urchins were severely affected by the storm (ca. 50% losses) with higher losses in those patches with a higher density of sea urchins. This may be due to a limited availability of refuges within each patch. In contrast, fish abundance was not affected, as fish were able to move to protected areas (i.e. deeper) as a result of the high mobility of this species. Our results highlight that catastrophic storms differentially affect the two dominant macroherbivores of rocky macroalgal and seagrass systems due to differences in mobility and escaping strategies. This study emphasises that under catastrophic disturbances, the presence of different responses among the key herbivores of the system may be critical for the maintenance of the herbivory function.

## Introduction

Most ecosystems are subject to disturbance regimes that operate across a range of temporal and spatial scales [1]. These disturbances, either by chronic (low energy, frequent) or catastrophic (high energy, infrequent) conditions are widely recognized as influencing the size, shape and abundance of many species in terrestrial and marine ecosystems [2]–[6]. Specifically, marine systems are particularly more prone to be regularly disturbed than many terrestrial settings due to the increased kinetic energy of the fluid medium in which they occur [7]. Hydrodynamic forces generated by waves are among the most important mechanisms of disturbance in coastal systems [8]–[10]. Indeed, extreme storm events expose most organisms to hydrodynamic forces that exceed their mechanical limits [2], and thus provide a mechanism for re-initiating ecological succession in disturbance-generated patches [11].

Depending on the spatial and temporal extent of the damage caused by the storm, the functioning and processes of ecosystems may be also importantly disrupted [1]. However, little is known on the effects of large infrequent disturbances on these functional aspects. Herbivory is a crucial ecological process in marine systems where it is known to contribute to the structure and organization of communities [12], [13]. In tropical environments, herbivory is responsible for maintaining coral reefs in a coral-dominated state [14], [15] and for exerting a great pressure on submersed vegetation, either on seagrasses or macroalgae [16]. In temperate environments, herbivory plays a central role as well, and it may induce shifts from macroalgal-dominated areas into coralline barrens, when herbivores are released from control by predators [17], [18]. Moreover, substantial effects of temperate herbivores have also been observed on seagrasses, such as the creation of mowed patches where seagrass primary production and canopy structure are severely altered [19], [20]. Due to its central role in structuring marine communities [12], disturbances affecting herbivory may profoundly influence underwater landscapes and associated ecosystem functions [e.g. 10]. A first step towards the understanding of how large infrequent storms may affect herbivory is to study the responses of key herbivores.

Animals are known to display contrasting responses to disturbances depending on their life-history traits [21]. Indeed, response diversity, the diversity of responses to environmental forcing among species that contribute to the same ecosystem function, appears to be particularly crucial when the system is subjected to disturbances [1], [22]. Compared to sessile organisms, the responses of mobile marine animals to disturbance have been less studied [but see for example 4,23,24], although extreme environmental conditions and physical forces can directly kill appreciable numbers of mobile marine animals [11]. Mobile animals can behaviourally avoid potentially lethal environmental stresses, an option not available to structural species. Highly mobile species can avoid the disturbance by actively moving to areas where hydrodynamic forces are less intense [6], [24]. This avoidance strategy will be successful or will fail depending on the rapidity with which harsh conditions develop and their intensity, as well as the degree of mobility of the organisms in question [11]. When mobility is limited, the effects of storms may be dependent on the number of refuges available in the habitat, and the capacity of the species to seek refuge [e.g. 25]. Whatever the individual response, each ecosystem will have a set of herbivores and the final effect of the disturbance on the herbivory process will be dependent on the responses of the key herbivores of the system, which in turn rely on their biological traits.

One classical example demonstrating the importance of different species displaying diverse responses in ecosystem resilience is that provided by Hughes (1994), reporting the well-known phase shift that occurred in the Caribbean coral reefs. In that case, overfishing sustained for decades reduced fish herbivores thus eroding response diversity in the functional group of grazers [1], [22]. However, their function (i.e. herbivory) was still maintained due to an increase in sea urchin grazing that preserved for a while a coral-dominated state. Nevertheless, when a species-specific pathogen plus a hurricane dramatically reduced the population of sea urchins, no other herbivore could compensate this loss and a shift to a macroalgal state took place. Beyond this example, the theoretical base supporting that having species that respond differently to disturbances can stabilize ecosystem process rates is strong [1], [22], [26], and this theoretical work has outpaced experimental work [but see 27], particularly in marine systems. Overall, there is a lack of studies assessing responses (particularly behavioural) to and recovery from a variety of disturbances [26]. This is especially true for mobile non-habitat forming key species, as they are more difficult to follow and generally do not form part of routine monitoring programs, which is how most disturbance effects are detected.

Herbivory is one of the main drivers of Mediterranean macrophyte communities. The two dominant herbivores, the low mobility sea urchin *Paracentrotus lividus* (Lam.) and the highly mobile fish *Sarpa salpa* (L.), have been identified as key organisms determining the organisation and functioning of macroalgal [28], [29] and seagrass communities [20], [30]. Both herbivores are browsers, although sea urchins may also be considered grazers in macroalgal systems (where they may induce coralline barrens [29]). As a first step towards the understanding of the potential effects of extreme storms on the herbivory process of Mediterranean macrophyte systems, we took advantage of a severe disturbance that occurred in December 2008, consisting on a violent and unprecedented storm. Our aims were (i) to test the response to the storm in terms of abundance of these two herbivores and (ii) to assess which were their escaping strategies. We hypothesised that each species would differ in its strategy depending on its degree of mobility. To address these objectives we used two parallel studies we were conducting before, during and after the storm. Specifically we assessed the responses in terms of abundance of both species, by monitoring fixed areas before and after the storm, and behaviour in fishes tagged with acoustic transmitters during the same period.

## Materials and Methods

### Ethics Statement

The Ethics Committee of the University of Barcelona issued a favourable report on the fish tagging protocol. The Department of Environment of the Catalan Government gives the permissions for fishing, operating and releasing the animals in the Medes Islands Marine Reserve. The reserve guards and the Spanish Marine Police (GEAS) supervised all operations.

### The Storm and the System

**Figure 1:**
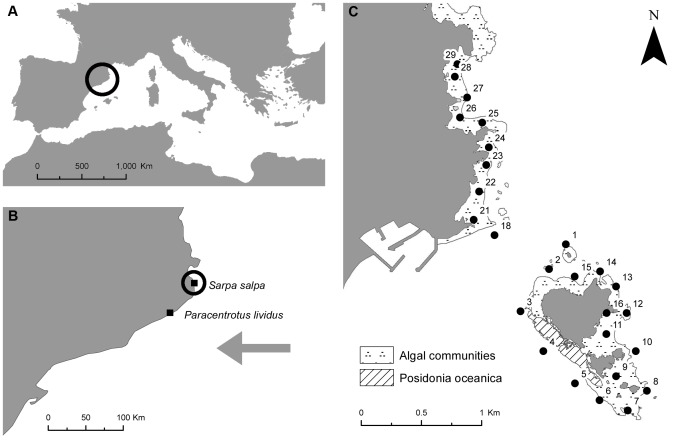
Location of the two monitoring programs conducted before and after the severe Easterly storm. Circled areas are zoomed in the next panel. The grey arrow in panel b) points out the direction of the storm (east). Panel c) shows the passive acoustic monitoring array deployed to track *S. salpa* individuals. Each receiver is numbered. This figure is composed of a topographic base map 1∶50 000 property of the Institut Cartogràfic de Catalunya (accessible from www.icc.cat) and a bionomic map property of the Universitat de Barcelona [60], [61].

The severe coastal storm that took place in the Catalan coast (NW Mediterranean) on 26–27^th^ December 2008 was a low frequency event with a returning period of more than 100 years. It hit with maximum winds of up to 20 m/s, significant wave heights as large as 8 m, record maximum wave heights in excess of 14 m, and wave periods of up to 14 s [31]. Damage to shallow coastal communities by currents and sand scouring during the storm is well documented after scientific scuba diving inspections [32]. Substantial reductions were observed on algal cover [33], populations of sea urchins [3], long-lived species of brown algae [34] and populations of gorgonians [35] in rocky substrates. In sandy bottoms, the storm strongly buried (>10 cm of burial) at least 20% of *Posidonia oceanica* (L.) Delile seagrass beds at depths less than 10 m and damaged and destroyed an unknown amount by abrasion, unearthing and uprooting of plants, which is particularly significant given the suspected very low recovery rate of this community [36]. The effects of this storm in terms of buried seagrass were visible to as deep as 23 m in some *P. oceanica* meadows. Our two study areas (see below) were severely affected by the storm, as they were included in the area where the storm energy was the highest (Fig. 1).

### Paracentrotus Lividus Assessment

Sea urchin population assessment was conducted in a shallow patchy *P. oceanica* meadow located on the Catalan Coast (N 41°41′ E 2°49′, Fig. 1), which had been monitored for 10 years and specifically 5 months prior to the storm event. The meadow is located at 8 m depth, in an open area exposed to east waves and currents, and therefore was importantly affected by the storm. The area boasts a large number of *P. oceanica* patches of different sizes and shapes. Each patch is separated from others by sand. In order to monitor these patches we marked 19 of them in summer 2008 with numbered iron bars.

Seagrass patch area before (2008) and after the storm (2009) was estimated by means of scaled photography. Photographs of each patch were taken from a zenithal point of view along with a known-length object (i.e. a 1.5 m iron bar). These pictures were then transferred to a computer where they were analysed in imageJ (v1.42q National Institutes of Health, USA). Each image was scaled using the known-length object and then the edge of each patch was traced in order to calculate the area. Additionally the percentage of buried, unburied and normal matte conditions was assessed visually for each seagrass patch few weeks after the storm. The same two divers, who had previously intercalibrated among themselves, made all estimations.

*P. lividus* total abundance (total number of individuals) per patch, density (number of individuals per square meter) and size distribution were obtained for the same 19 patches of different sizes (i.e. from <0.5 to 5 m^2^) before and after the storm. This was done by exhaustive visual inspection and careful, repeated introduction of bare hands among the rhizomes to allow sensing of hidden individuals [37]. For every urchin counted, its horizontal test diameter without spines (TD) was measured to the nearest mm with a calliper. The same protocol was used before (i.e. summer-autumn 2008) and after the storm (i.e. spring-summer 2009). Assuming that (i) our sampling method is able to effectively detect urchins >1 cm (test without spines); (ii) that adult *P. lividus* populations are very homogeneous and relatively stable over long periods of time [38], [39]; and that (iii) migration is unlikely between seagrass patches in the study site owing to their being in a sand matrix and separated by some meters one another [39], we can compare populations in each patch before and after the storm with a high confidence that the urchins lost were gone as a consequence of the storm.

### Sarpa Salpa Assessment

*S. salpa* abundance sampling was also carried out in the Catalan coast, in Medes Islands Marine Protected Area and its neighbouring unprotected coast (N 42°3′, E 3°13′; Fig. 1b,c) during the years 1999, 2002, 2005 (before the storm), 2009 (after the storm), 2011 and 2012. Abundance was assessed by scuba diving over 50×5 m (250 m^2^) transects haphazardly placed in 3 zones within the study area between 5 and 10 m depth. All *S. salpa* fish in each transect were counted. A total of 136 transects were sampled between 1999 and 2012. Sampling was always conducted during summertime (from August to September) in order to avoid seasonal differences among years.

An acoustic monitoring programme to estimate movement patterns of *S. salpa* fishes started 2 months prior to the storm and ended 10 months after. It was also carried out in Medes Islands Marine Protected Area and its adjacent unprotected stretch of coast (N 42°3′, E 3°13′; Fig. 1b,c). There, a fixed array of receivers (VEMCO, VR2 receivers, Nova Scotia, Canada) was deployed around the islands and along the coast (Fig. 1c). Receivers’ detection range was established at 250 m prior to fish tagging. The average distance between receivers was 210 m. Receivers were retrieved, data downloaded, cleaned of biofouling, and redeployed 5 times during the study (in November 2008, January 2009, May 2009, August 2009 and October 2009).

*S. salpa* individuals were fished and tagged on the 16^th^ and 17^th^ October 2008. Twenty fishes were captured at four different sites using seine-fishing nets. Following capture, fishes were transferred to an anaesthetic bath of 0.2 ml l^−1^ 2-phenoxyethanol [40]. When each fish was immobilized (showing no reaction to external stimuli) they were placed on a V-shaped support. Incision area was de-scaled and an incision approximately 2 cm long was made between the anal fin and the anus. An acoustic transmitter (VEMCO, V9P-2L, 9 mm diameter ×47 mm length, 120 s average repeat rate, 522 days of estimated battery life, ±2.5 m depth accuracy) coated in antiseptic was inserted into the peritoneal cavity. The incision was closed using a sterile surgical stapler (3–4 staples). Fishes were placed in a monitoring bucket where their gills were flooded with fresh seawater until they regained equilibrium. Subsequently, they were kept in an underwater cage until complete recovery, and then they were released to their respective fishing-sites. The full procedure (from anaesthesia to initial recovery) took around 7 min. Previous studies have shown that surgical tag implantation has a very limited impact on the behaviour and physical status of this species [41]. From the 20 fishes tagged, 16 fishes were lost before the storm event, and only four were still being continuously tracked around that time (26–27^th^ December 2008). These are the only ones that are analysed here.

### Data Analysis

Two dependent variables were assessed from the sea urchins’ data set. Sea urchin abundance per patch and number of sea urchins lost per patch. Regarding sea urchin abundance, a General Linear Model (GLM) with a Negative Binomial distribution (and a logarithmic link function) was used to test the significance of the explanatory variables. Negative binomial distributions are generally used to deal with count data with overdispersion [42], as it was the case. We introduced into the model ‘time’ (2 levels: before and after the storm) and sea urchin ‘size’ (6 levels: 1–2] cm, 2–3] cm, 3–4] cm, 4–5] cm 5–6] cm and >6 cm test diameter) as fixed factors and ‘patch area’ and ‘% of unburied matte’ (rhizome layer exposed) as fixed continuous variables. A preliminary exploration identified collinearity among the variables % of unburied matte (rhizome layer exposed), % of buried and % normal matte conditions. Thus, only % of unburied matte was entered into the model. On the other hand, the dependent variable number of sea urchins lost per patch was analysed with a GLM with a Poisson distribution (and a logarithmic link). We wanted to test whether there were differences in the number of sea urchins lost according to the fixed explanatory factor sea urchin ‘size’ (factor with 6 levels, see above), and the continuous variables ‘sea urchin density before the storm’, ‘% of seagrass area lost as a consequence of the storm’ and ‘% of unburied matte’. Since the number of sea urchins lost per patch depended on the abundance of sea urchins per patch, this variable was included as an offset [42]. In this way, we modelled the effect of explanatory variables on the ratio between the number of sea urchins lost per patch and the abundance of sea urchins per patch. In all cases, the best models were selected based on the Akaike’s Information Criterion (AIC) [42]. Data were checked for normality by the visual inspection of plots of residuals and fitted values. All data were analysed with the packages MASS [43] and stats in the statistical software R [44].

Regarding *S. salpa*, we also modelled their abundance (from the visual transects) as a function of the fixed factors ‘year’ (6 levels: 1999, 2002, 2005 [before the storm], 2009 [after the storm], 2011 and 2012) and ‘zone’ (3 levels, the 3 sampled zones). Given that the data set was again count data with overdispersion, we used a GLM with a negative binomial distribution (and a logarithmic link). We were also interested in knowing whether the movement behaviour of tagged fish was related to the storm event. To this end we correlated for each fish (n = 4) its daily mean depth and its total distance travelled per day with the daily maximum wave height obtained at the same site (kindly provided by Josep Pascual, from l’Estartit Observatory N 42°3′, E 3°12′). Fish depth is one of the parameters that fish transmitters provide, but total distance travelled per fish was calculated from the fish detections among different receivers with the package adehabitaLT in R [45]. These distances should be viewed with caution and as a comparative measure between days and fishes. It should be borne in mind that the distances calculated do not derive from the actual fish trajectory but from the receivers that detect a given fish at a given time (e.g. if a fish is continuously moving on the periphery of the detection range between two receivers, it would actually move some tens of meters but since it would have been detected by different receivers, the estimated distance would be much higher). All time series were detrended, if necessary, by regressing each of them with time. If the regression was significant (i.e. a trend was found), the residuals were used in further analyses. Normality of the time series was checked before calculating regressions and if not fulfilled, time series were transformed. Finally, the cross-correlation coefficients (Pearson) between each fish time series and the daily maximum wave height were calculated at different time lags. All calculations were performed in R.

## Results

### Paracentrotus Lividus Assessment

**Figure 2:**
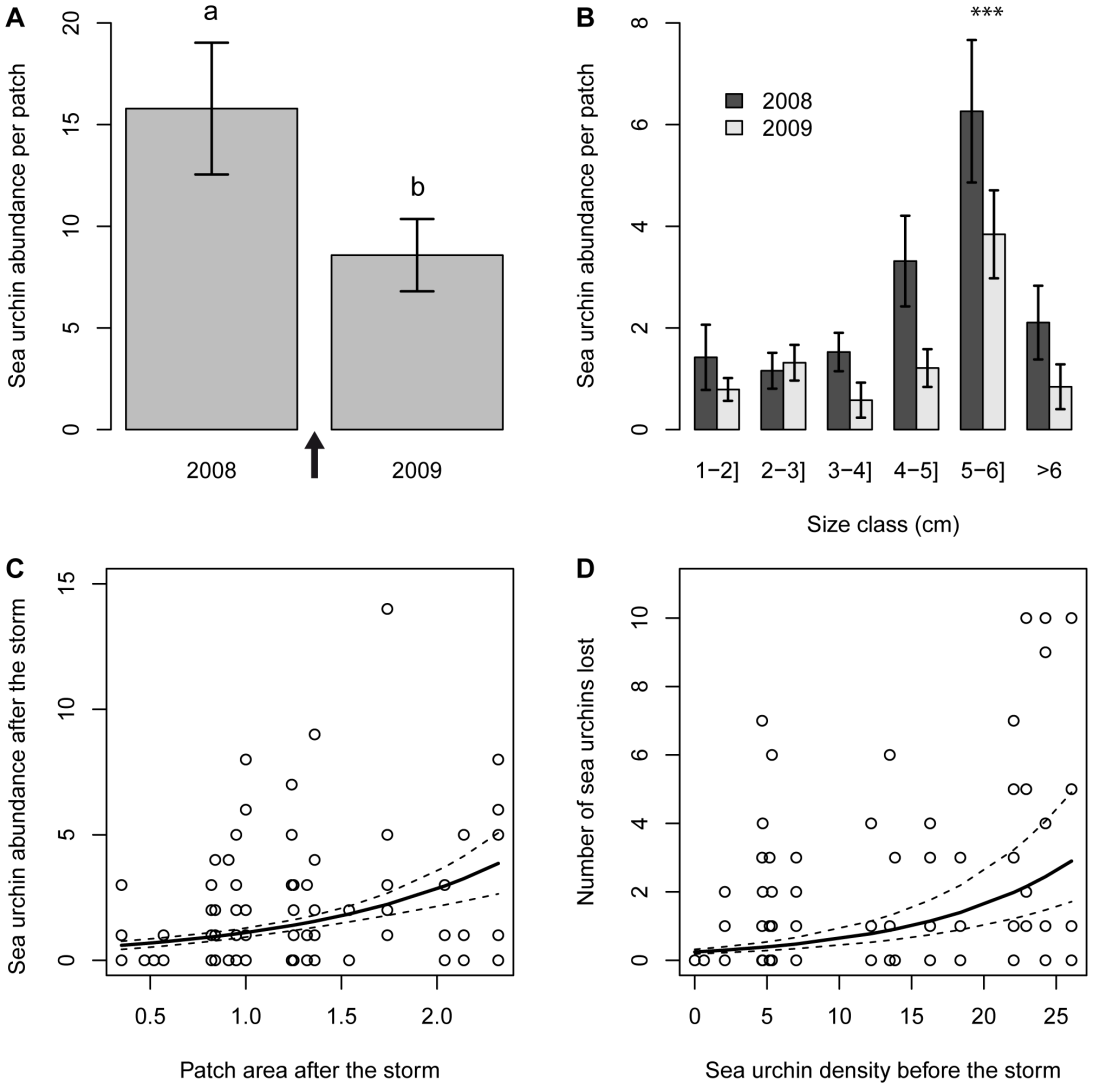
Effects of the storm on *P.* lividus population. a) Effect of the factor time (before the storm [2008] and after the storm [2009]) to the mean sea urchin abundance per patch. Different lower case letters indicate statistically significant differences (see Table 1). The arrow symbolizes the storm event. b) Effect of the factor size and time on mean sea urchin abundance per patch. Note that sea urchins of 5–6 cm were the most abundant; the asterisks show this was significant (Table 1). c) Effect of seagrass patch area after the storm on the abundance of sea urchins per patch after the storm. d) Effect of sea urchin density before the storm on the number of sea urchins lost per patch. Model fits are plotted as solid lines ± the standard error (see model coefficients in Table 1).

The Generalised Linear Model for sea urchin abundance revealed that the factor time, i.e. the storm, had a great effect on the abundance of sea urchins per patch (Fig. 2a, Table 1). Indeed, total sea urchin abundance (adding all patches) before the storm was 280 individuals, whereas after the storm total abundance had decreased to 145 individuals, resulting thus in a loss of 48% of sea urchins. The 5–6 cm size-class was significantly more abundant compared to the others (Fig. 2b, Table 1). However, sea urchin loss was similar across all size classes (Fig. 2b), as the interaction time × size was not significant, and was dropped from the best-selected model. Sea urchin abundance was also significantly dependent on patch area (Table 1). However, the relationship between patch area and sea urchin abundance was influenced by the factor time, as indicated by the significant time × area interaction. This may be related, on the one hand, to the fact that the storm affected the area of seagrass patches, making them shrink in size. And on the other, to the fact that before the storm smaller patches exhibited higher sea urchin abundance, i.e. were denser than bigger patches; while after the storm smaller patches displayed lower sea urchin abundance (Fig. 2c). These results agree with the significant effect of sea urchin density before the storm on the dependent variable number of sea urchins lost (Table 1, Fig. 2d). This means that the denser the sea urchin population in the patch, the more sea urchins were lost per patch as a consequence of the storm (Fig. 2d). The % of patch area lost because of the storm had also a significant influence on the number of sea urchins lost (Table 1). However, the effect of this parameter was limited (see the low coefficient in Table 1). Finally, sea urchin loss was independent from the % of unburied matte or sea urchin size, since these variables were dropped from the model during model selection.

**Table 1:** Sea urchin data analysis.

Dependent variable	Effect	Coefficient (SE)	z-value
**Sea urchin abundance**	Intercept	11.1 (3.3)	3.4 ***
	Time	−1.4 (0.4)	−3.5 ***
	Area	−5.1 (2.1)	−2.5 *
	Size 1–2]	−0.1 (0.3)	−0.3
	Size 2–3]	0.1 (0.3)	0.2
	Size 3–4]	−0.2 (0.3)	−0.6
	Size 4–5]	0.5 (0.3)	1.5
	Size 5–6]	1.4 (0.3)	4.4 ***
	Time × Area	0.7 (0.3)	2.6 **
**Sea urchins lost**	Intercept	−1.8 (0.3)	−6.5 ***
	Density 2008	0.04 (0.01)	3.6 ***
	% of patch area lost	0.01 (0.00)	2.7 **

Significance codes: 0***, 0.001**, 0.01*, 0.05 ·, 0.1. SE: Standard Error.

Generalized Linear Models (GLMs) showing the effect of explanatory variables to sea urchin abundance and number of sea urchins lost after the storm. The table shows the best-selected models with parameter estimates. The best selected models were a GLM with a negative binomial distribution (and a logarithmic link function) for sea urchin abundance; and a GLM with Poisson distribution (and a logarithmic link function, Φ (dispersion) = 0.81) for sea urchins lost.

### Sarpa Salpa Assessment

**Figure 3:**
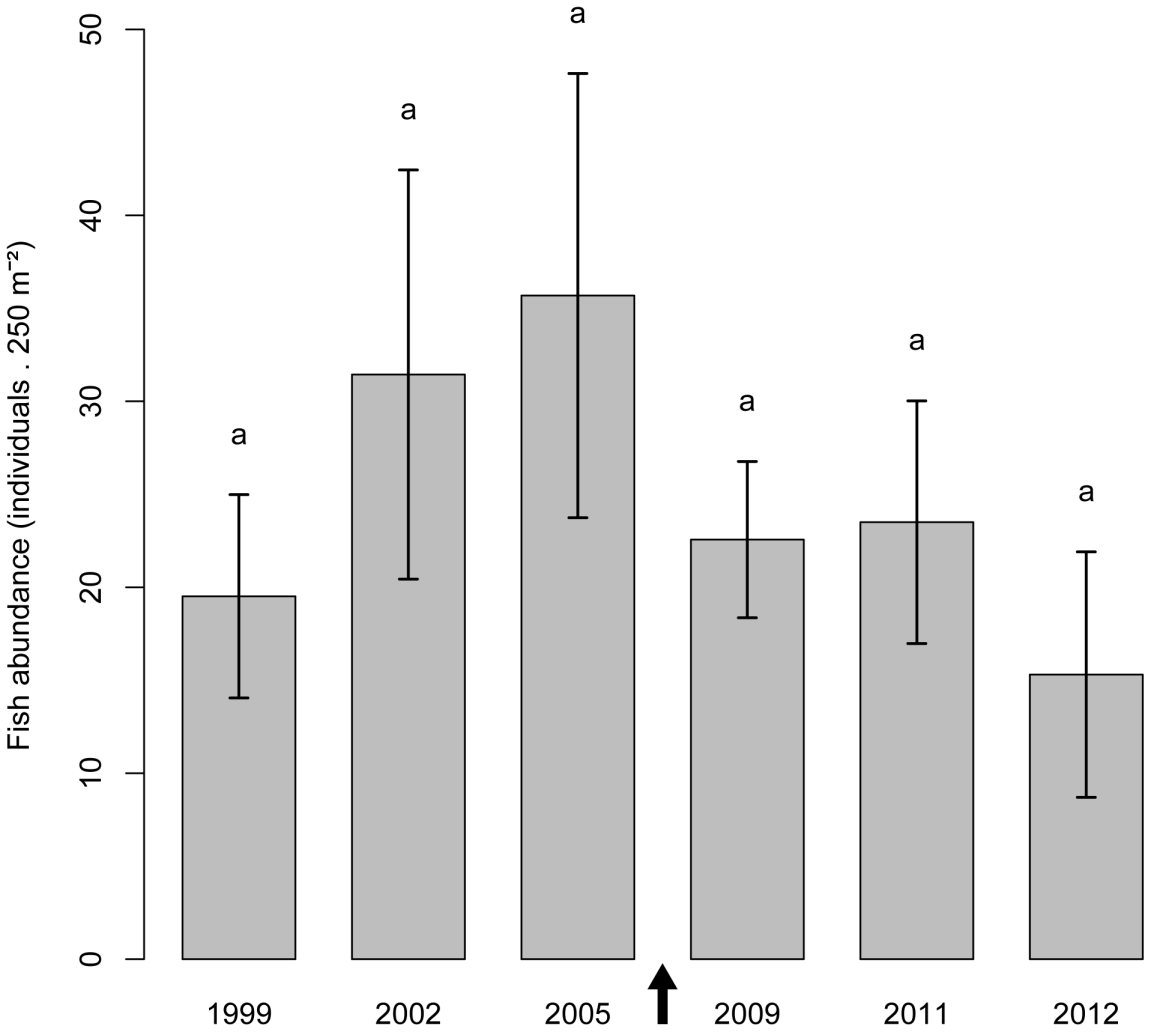
Effects of the storm on *S. salpa* population. Mean *S. salpa* abundance per transect (number of *S. salpa* individuals per 250 m^2^) as a function of the factor year. The same lower case letters indicate that differences were not statistically significant (see Table 2). The arrow indicates the storm event (December 2008).

**Figure 4:**
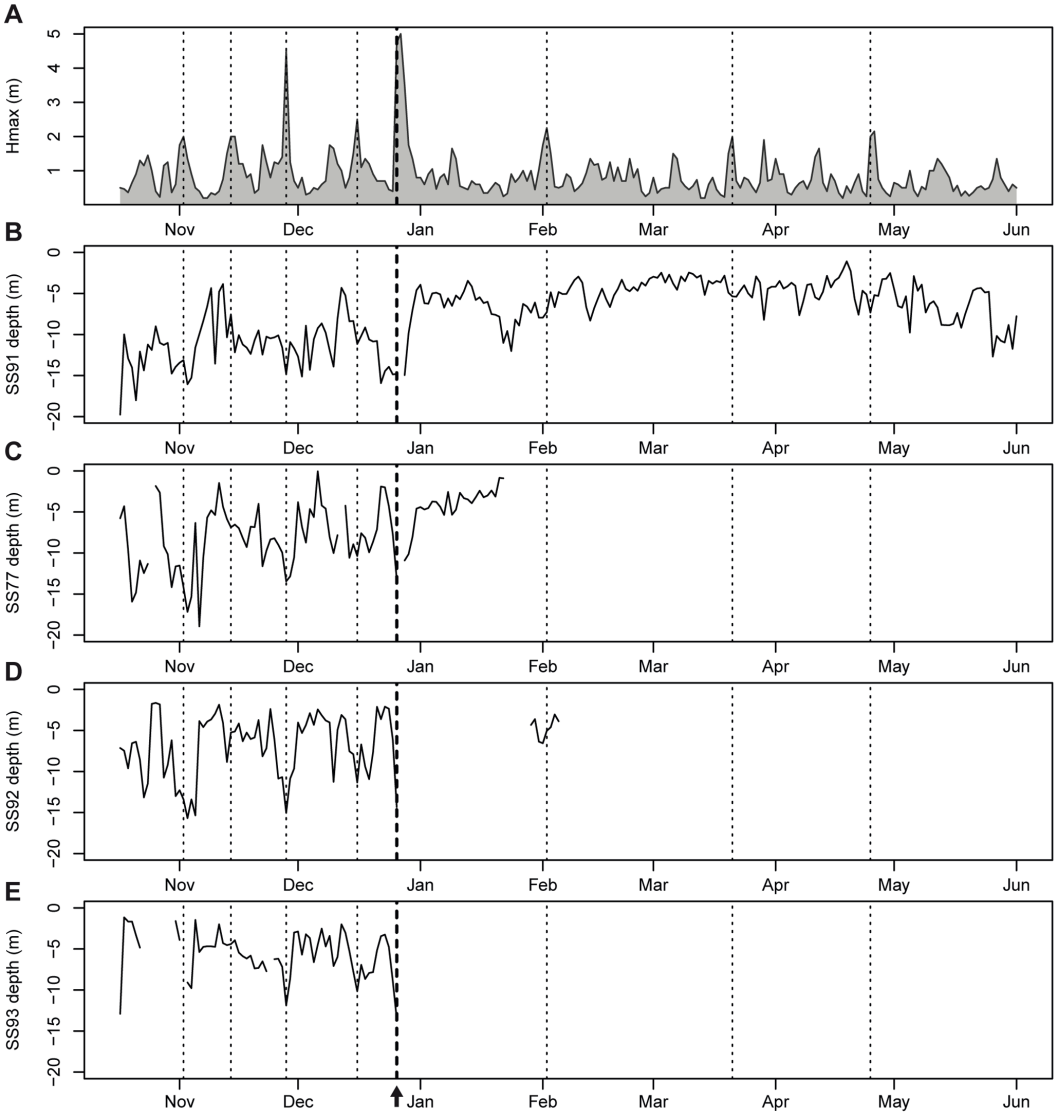
Time series of the daily maximum wave height (Hmax) and *S.*salpa daily mean depth. Each panel corresponds to an individual fish. Vertical dotted lines indicate the date of storms with Hmax >2 m, and the thicker dashed line with an arrow indicates the date of the December 2008 catastrophic storm event. Note that fishes responded to most storms by moving to deeper areas. Note the disappearance of some fishes for several days following the studied storm.

**Figure 5:**
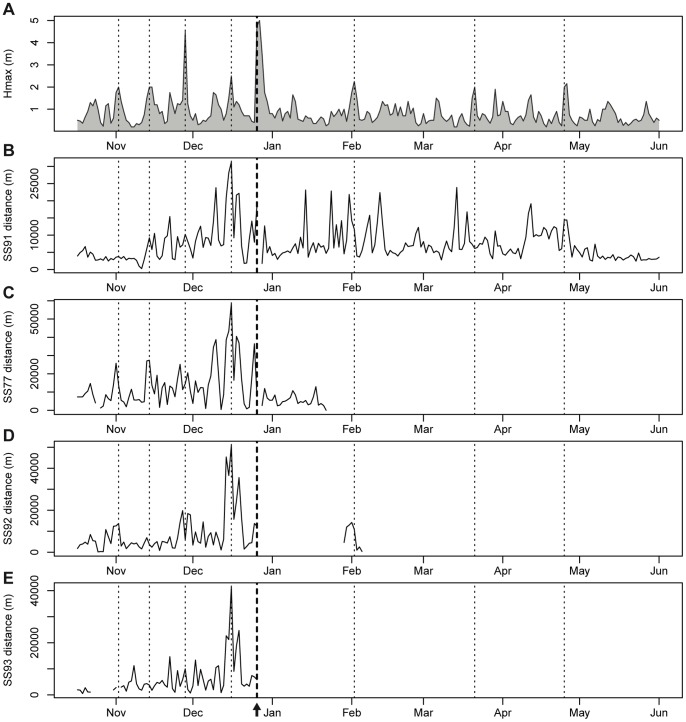
Time series of the daily maximum wave height (Hmax) and *S.* salpa travelled distanced per day. Each panel corresponds to an individual fish. Vertical dotted lines indicate the date of storms with Hmax >2 m, and the thicker dashed line with an arrow indicates the date of the December 2008 catastrophic storm event. Note that in general the distances travelled per day were higher in stormy days.

In contrast to the sea urchin population, *S. salpa* abundance was not influenced by the storm, as shown by the non-significant year effect (Fig. 3, Table 2). In contrast, fish behaviour did respond to the severe storm of December 2008 (see Fig. 4a to note that this storm was the heaviest of the whole time series), as well as to other important storms along the tracking period (see Figs. 4, 5). Indeed, all fish individuals presented a depth distribution that was significantly and negatively correlated with the time series of maximum wave heights (Hmax) (Table 3, Fig. 4). This means that fishes moved to deeper areas on stormy days (Hmax and *S. salpa* daily mean depth time series were on phase, see Table 3). In addition, the distance travelled per day was also significantly correlated with Hmax (except for fish SS93), but positively in this case (Table 3, see Fig. 5). Thus, fishes made longer trips on stormy days (both time series were on phase). In fact, in some cases fishes moved from their core areas (mostly receiver 4, see Fig. 1c) to distant zones even outside our receiver array (see blank spaces in the time series of fish SS92 and SS93, Fig. 5d,e). Some fishes rapidly returned to their core areas few days after the storm (Fig. 5b,c), while others returned after some months (Fig. 5d), and one never returned (Fig. 5e).

**Table 2:** *S. salpa* abundance analysis.

Term dropped	df	AIC	Likelihood ratio test	p-value
<None>	13	1034		
Zone × Year	9	1031	4.8	0.3
Year	4	1024	3.1	0.7
Zone	7	1027	0.2	0.9

Results of *S. salpa* abundance model selection with a Generalized Linear Model (GLM). The selected family distribution for the GLM was a negative binomial (with a logarithmic link function) owing to the overdispersion present in the data set. We present the significance of dropping each variable one by one from the full model. The full model was Abundance ∼ Zone+Year+Zone × Year. Akaike’s Information Criterion (AIC) was used to select the best model, evaluating the trade-off between model parsimony and goodness of fit. The lower the AIC the better the model.

**Table 3:** *Sarpa salpa* time series analysis.

Comparison	Ts. length	Lag	Coefficient	Fish ts. filtering
Hmax – SS77_depth	95 d	0 d	−0.52 ***	Detrended
Hmax – SS91_depth	228 d	0 d	−0.27 **	Log and detrended
Hmax – SS92_depth	79 d	0 d	−0.58 ***	Log and detrended
Hmax – SS93_depth	61 d	0 d	−0.33 **	Log and detrended
Hmax – SS77_distance	98 d	0 d	0.39 ***	Sqrt
Hmax – SS91_distance	228 d	0 d	0.19 **	Log
Hmax – SS92_distance	79 d	0 d	0.40 **	Sqrt and detrended
Hmax – SS93_distance	62 d	–	n. s.	Log and detrended

Sqrt: square root transformation; log: log-transformation. Significance codes: 0***, 0.001**, 0.01*, 0.05 ·, 0.1.

Pearson correlation coefficients between the time series of individual fishes and of the daily maximum wave heights (Hmax). The first column indicates the time series that are being compared. The second column indicates the duration of the time series analysed. We then indicate the lag, in days, at which the correlation coefficient was maximal, and these coefficients. We finally specify the treatment applied to each fish time series, which were transformed and/or detrended if necessary. Hmax time series was always log-transformed and detrended, since it did not fulfil normality or stationarity.

## Discussion

As expected from their very different movement capacities, both herbivores responded differently to the extreme storm event. In terms of abundance, the mobile species (the fish *S. salpa*) endured the disturbance with non-significant losses, while nearly half of the population of the least mobile species (the sea urchin *P. lividus*) was wiped out by the waves in seagrass meadows. The higher survival observed for the fish population could be the result of their active escaping strategy, consisting on sinking to deeper waters or on moving to other areas. In contrast, the sea urchin could only seek shelter within the seagrass habitat, which appears to give a moderate protection against currents and waves, at least in such extreme events. These findings suggest that, after the catastrophic storm, herbivory by fishes may remain more or less unaffected, while herbivory by sea urchins may substantially decrease without disappearing. This is particularly relevant in Mediterranean seagrass and rocky systems, given their low number of species within the macroherbivorous group.

*P. lividus* cannot move great distances [39], [46], and therefore, their only possible mechanism to withstand a storm is by using the habitat for shelter. Sea urchins have been observed to decrease their ability to forage even in low hydrodynamic regimes (much lower than their dislodgement forces) [4] and to hide in crevices and decrease their movement rates when water turbulence increases [23], [39]. In spite of these behavioural adjustments (i.e. decreased movement rates, shelter-seeking behaviour), under extreme storm events sea urchin populations are generally importantly affected [10], [47], [48]. In this study, the seagrass-dwelling population of sea urchins was also greatly affected by the storm, with half of the population swept away from the studied seagrass patches. Our results suggest that shelter-seeking behaviour could have attenuated sea urchin losses on those patches with available refuges, since patches displaying a higher density of sea urchins before the storm (putatively with less refuge availability) lost more individuals during the event. *P. oceanica* is known to provide sea urchins with shelter from predation within the root-rhizome layer [37], [49], and it is very likely that they are also using this complex structure to escape from increased water movement. We also expected a significant effect of size on the number of sea urchins lost. Bigger sea urchins are more prone to be dislodged, given that the force due to water's acceleration increases faster than the organism's structural strength as the organism grows [50]. However, this was not the case in the present study, most probably due to the attenuating effect of the seagrass [7]. In contrast, in a much less structured habitat (rocky bottom with boulders) the same storm caused sharper sea urchin abundance decreases (65% on average [3], compared to 48% in the seagrass), with a significant size effect. In that case, since algal canopies provide less protection than seagrass canopies (specially compared to the large *P. oceanica* seagrass), smaller sea urchins were still able to find shelter, but larger ones could not find suitable refuges and were nearly all lost (80–100% of individuals above 3 cm [48]). Thus, our results suggest that being inside a seagrass canopy gives better chances of surviving an extreme storm than being in an algal-covered rocky habitat.

The escaping strategy used by *S. salpa* appeared to be more successful and was in clear contrast with that of sea urchins. Few *S. salpa* individuals were found stranded on the beaches after the storm and no sound effects on their population were apparent in the long-term data series of *S. salpa* abundance (Fig. 3). This is in accordance with the fact that at least three out of the four fishes tagged survived the storm, while we cannot attribute the disappearance of the fourth to mortality, since it could have relocated to areas out of our receiver array. These herbivorous fishes actively moved to deeper, more protected waters the day of the storm, which presumably reduced the probability of being swept ashore by large waves as well as mitigated the potential effects of mechanical damages from suspended sand and other debris [6]. Indeed, fishes responded by moving to deeper areas in most of the storms observed during the studied period, as shown by the negative correlation found between maximum wave height and *S. salpa* daily mean depth (Fig. 4, Table 3). In addition, stormy days were days of long distance movements (positive correlation between wave height and distance moved, Table 3, Fig. 5), since fishes presumably moved from their core areas (seagrass meadow area, see Fig. 1c, Pagès et al. unpublished data) to more protected zones. This has been reported after hurricanes for coral reef fish species [24], [51], [52], and sometimes has been attributed to the effect of currents.

The contrasting responses of both key herbivores clearly points out that mobility patterns can be fundamental to understand species-specific responses to catastrophic storm events. As seen, each of these herbivores operates at a different scale. Sea urchins escaped taking advantage of the structure offered by the seagrass habitat, and at the most making movements on the meter scale (i.e. moving deeper into the root-rhizome layer of the seagrass, moving towards nearby crevices, etc.) due to their small home range [46], [53]. On the other hand, *S. salpa* escaped moving hundreds of meters or even some kilometers (Figs. 4,5) given its large home range [40,54, Pagès et al. unpublished data]. It may not be surprising that two herbivorous species so profoundly different in many traits (an echinoderm and a fish) show differential responses when faced with disturbances. However, for the studied storm, which was particularly extreme and with a returning period of a 100 years [31], many other species of fishes were profoundly affected. In the studied zone, beaches were completely covered with stranded fishes of several species, but not *S. salpa* (personal observations). Additionally, a parallel project in our study area assessing the effects of the same storm on other fish populations, found that *Anthias anthias* (L.) and *Chromis chromis* (L.), which display a more site-attached behaviour, were severely affected and were swept ashore in great numbers [55]. This has also been observed in other studies that have pointed out that more mobile fish species are generally less affected by physical disturbances than sedentary ones [6], [56]. So, more than the phylogenetic position of the species what seems crucial is the species’ movement behaviour in relation to the disturbance. Given that disturbances generally operate across a limited range of scales, animals that can respond across different scales may be better suited to withstand a variety of disturbances [1]. Similarly, if different species in a functional group operate at different scales, they may provide mutual reinforcement contributing to the resilience of the function, while at the same time minimizing competition among species within the functional group [1], [57]. This is known as scale range redundancy and may be occurring within the Mediterranean herbivorous group, given the differences in mobility and behaviour.

Response diversity within functional groups has been suggested to be of crucial importance to ensure the resilience of ecosystem functions [1]. While our study did not assess whether the function provided by *S. salpa* and *P. lividus* (i.e. herbivory) changed as a result of the disturbance, it is known that both herbivores are important functional elements [30], [58], [59]. In our system, given the low number of herbivorous species, herbivory function would be easily eroded should both herbivores respond in a similar way to disturbances. However, we have shown that even faced with large infrequent disturbances such as the 2008 storm, at least one of the herbivorous species of the system would be able to maintain the herbivory function.
